# Mutation on JmjC domain of UTX impaired its antitumor effects in pancreatic cancer via inhibiting G0S2 expression and activating the Toll-like signaling pathway

**DOI:** 10.1186/s10020-024-01023-2

**Published:** 2024-12-20

**Authors:** Xiao-hua Shen, Shu-ping Xiong, Sheng-peng Wang, Shan Lu, Yi-ye Wan, Hui-qing Zhang

**Affiliations:** 1 Department of Gastrointestinal Medical Oncology, Jiangxi Cancer Hospital, Nanchang, Jiangxi 330029 People’s Republic of China; 2 The Second Department of Radiotherapy, Jiangxi Cancer Hospital, Nanchang, Jiangxi 330029 People’s Republic of China; 3 The Medical College of Nanchang University, Nanchang, Jiangxi 330006 People’s Republic of China; 4 JXHC Key Laboratory of Tumor Microenvironment and Immunoregulation (Jiangxi Cancer Hospital), Nanchang, Jiangxi 330029 People’s Republic of China

**Keywords:** Pancreatic cancer, UTX, H3K27me3, G0S2, Toll-like signaling pathway

## Abstract

**Background:**

Recently, the incidence of pancreatic cancer (PC) has gradually increased. Research has shown that UTX mutants are critical in tumors. However, the underlying mechanisms remain incompletely understood. This study aimed to explore how UTX mutation would affect its related function in PC.

**Method:**

Exome sequencing was used to analyze PC samples. MTT, transwell, and colony formation assays were performed to determine the cellular functions of PC cells. qRT-PCR, Western Blot, TUNEL, immunohistochemistry, CHIP, bioinformatics, and xenograft experiments were used to investigate the mechanism of UTX mutants in PC in vitro and in vivo.

**Results:**

We compared exome sequencing data from 12 PC samples and found a UTX missense mutation on the JmjC structure. Through cellular functions and xenograft experiments, wild-type UTX was found to significantly inhibit PC malignant progression in vitro and in vivo, while UTX mutation notably impaired this effect. Furthermore, G0S2 was identified as the key target gene for UTX, and wild-type UTX significantly increased its expression, while mutant one lost this function to a certain extent both in vitro and in vivo. More importantly, G0S2 overexpression not only inhibited tumor malignant phenotype and drug resistance for Gemcitabine in PC but also effectively reversed the roles of UTX mutant with Toll-like signaling pathway involved. In terms of mechanism, UTX mutation elevated the H3K27me3 modification level of the G0S2 promoter, which decreased its expression in PC cells.

**Conclusion:**

In conclusion, UTX mutant weakened the antitumor effect of wild-type UTX in PC by inhibiting G0S2 expression and activating the Toll-like signaling pathway.

**Supplementary Information:**

The online version contains supplementary material available at 10.1186/s10020-024-01023-2.

## Introduction

Pancreatic cancer (PC) is a deadly malignancy since there are no significantly early symptoms and it can rapidly spread to other organs. Recently, the incidence of PC has increased and accounts for 5% of cancer-related deaths (Goral 2015). Several groups identified that PC may result from smoking, diabetes, obesity, genetics, etc. (Torphy et al. 2020). The major treatment of PC is surgical resection and chemotherapy (including adjuvant therapies: gemcitabine, capecitabine, etc.), but they’re often associated with poor prognosis, with an overall 5-year relative survival rate of about 10% (Huang et al. 2021). Therefore, there is an urgent need to find biomarkers for early diagnosis and therapeutic targets for PC.

Recently, researchers have shown that histone posttranslational modifications, a major epigenetic mark, play a critical role in tumors (Hogg et al. 2020). The abnormal histone methylation levels, including the imbalance of H3K27me3 methylation, may lead to oncogene activation and cellular carcinogenesis. Notably, studies by Zhou showed that the low level of H3K27me3 inhibits the malignant progression of PC, which provides a direction for the treatment of PC (Zhou et al. 2020). Ubiquitously transcribed tetratricopeptide repeat, X chromosome (UTX)/KDM6A, as a highly mutated oncogene, has been described to be a histone demethylase in several cancers (pancreatic cancer, breast cancer, and acute myeloid leukemia). UTX, encoded by the X chromosome but can escape X chromosome inactivation, contains one JmjC structure and multiple TPR structures and functions as a tumor suppressor gene or oncogene depending on the tumor type and context. Several studies have shown that UTX can act as a tumor suppressor to promote gene expression and cell cycle regulation by removing methylation modifications of H3K27me3. In addition, UTX can interact with tumor suppressors such as p53 to enhance its ability to inhibit tumor growth and spread (Wang et al. 2010; Kaneko and Li 2018; Shi et al. 2021). The domain of JmjC has been proven responsible for the H3K27me2/3 demethylase activity of UTX. Shi et al. suggested that the UTX mutation disables the ability to fight off cancer (Shi et al. 2021). Studies found that the high expression of UTX in colon cancer tissue (Tang et al. 2019), while the loss of UTX led to malignant progression of lymphoma and UTX mutations affected the drug sensitivity of cytarabine (Li et al. 2018). Meanwhile, the mutation rate of UTX is high in tumor tissues by exon sequencing analysis, which may be an important factor for the malignant progression of many tumors. However, there are also some studies suggesting that UTX can also act as a carcinogenic protein in some cases. For example, demethylase inhibition has antineuroblastoma activity (Lochmann et al. 2018). UTX knockdown significantly reduces breast cancer cell proliferation and invasiveness in vitro and in a mouse xenograft model (Kim et al. 2014). In addition, UTX can inhibit tumor cell apoptosis by removing the methylation modification of H3K27me3, thus promoting tumor growth and spread (Cottone et al. 2020). Therefore, the role of UTX as a tumor suppressor and carcinogenic protein is context-dependent and requires in-depth study and interpretation in terms of specific disease types and molecular mechanisms. Interestingly, our early study found that the loss of UTX is associated with the poor prognosis of PC. The loss of GATA6-mediated up-regulation of UTX promoted PC progression (Zhang et al. 2024). Additionally, Jaclyn Andricovich et al. found that UTX not only plays a role in H3K27me3 demethylation in PC but also is involved in regulating the activation of several signaling pathways (Andricovich et al. 2018). Nevertheless, the precise mechanisms underlying how the mutation and loss of UTX contribute to oncogene activation remain incompletely understood.

In this study, we identified mutationsUTX in the JmjC domain in PC through exome sequencing. We aim to elucidate the underlying mechanism of mutant UTX in PC. A comprehensive understanding of how UTX mutant affects histone modifications will provide valuable insights for the development of novel and effective treatments for PC.

## Materials and methods

### Exome sequencing analysis

To determine the mutation of UTX, exome sequencing was performed in 12 pancreatic cancer samples (cancer tissue: experimental group; paracancerous tissue: control group)) that were collected from Jiangxi Cancer Hospital. All the patients signed an informed consent in this research and didn’t accept any neo-adjuvant treatment before surgery. This research was approved by the Ethics Review Committee of Jiangxi Cancer Hospital.

### Cells culture

The PC cell lines PANC-1, BxPC-3, AsPC-1, and SW1990, obtained from INDIT Bio-Technology Co., Ltd (Hangzhou, China), were used. PANC-1 cells were cultured in DMEM medium (INDIT Bio-Technology Co., Ltd) containing 10% fetal bovine serum (FBS, SERANA, Brandenburg, Germany, S-FBS-SA-015) and 1% penicillin/streptomycin (PS, Gibco, Semitic, NY, USA, 15,140–122), while BxPC-3, AsPC-1, and SW1990 cells were cultured in RPMI-1640 medium (Tecono, Hangzhou, China) with the same supplements. Both cell lines were incubated at 37 °C with 5% CO2 in a humidified incubator (Thermo Fisher, Waltham, MA, USA, NO. 311).

### Cell transfection

The wild-type UTX (UTX-Wt), mutant UTX (UTX-Mut), and overexpressed G0S2 lentiviruses as well as the control one were constructed by Guannan Biological (Hangzhou, China). 100 μL reconstructed lentivirus (1 × 10^8^ Tu/mL) were added separately to PANC-1 and BxPC-3 cells, which were seeded separately in 24-well plates. After infection for 72 h, the cells were screened by 0.2 μg/mL puromycin (Thermo Fisher, A1113802), and the overexpressed effect was verified by the WB experiment.

UTX-Wt, UTX-Mut and G0S2 overexpression plasmids were acquired from Youbio(Hunan, China) and cloned into pcDNA3.1 vector to obtain the reconstructed-overexpressed plasmids. Small interfering RNA targeting G0S2 (siR-G0S2) and negative control siRNA (siR-NC) plasmids were also obtained from Youbio for knockdown experiments. PC cells were transfected with these plasmids using Lipofectamine 2000 at 80% confluence, respectively, and the transfection efficiency was assessed by WB. siRNA sequences are as follows:

siR-NC sense: UUCUCCGAACGAGUCACGUTT.

siR-NC antisense: ACGUGACUCGUUCGGAGAAT.

siR-G0S2 sense: GAUGGUGAAGCUGUACGUGCU.

siR-G0S2 antisense: AGCACGUACAGCUUCACCAUC.

### Cells proliferation assay

The proliferation capacity of PANC-1 and BxPC-3 cells was determined by MTT assay. UTX-Wt and UTX-Mut overexpressed as well as the corresponding control cells were seeded separately into 96-well plates (5 × 10^3^ cells/well) to culture at 37℃ for 0, 24, 48, and 72 h. Then 50 μL/well MTT (1 mg/mL, Sigma Aldrich, USA, M2128) was added. After cultured for 3 h, DMSO (150 μL/ well) was added and absorbance at 570 nm was also detected with the mixed solution. The procedures of drug resistance detection for gemcitabine, capecitabine, and oxaliplatin were consistent with the above steps.

### Transwell assay

Transwell assay was used to determine cell migration and invasion abilities. The procedure of the migration experiment was as follows: cells (5 × 10^4^ cells/well) were resuspended with serum-free medium and seeded in a transwell chamber, placed in a 24-well plate filled with 500 μL medium containing 10% FBS. After incubating for 24 h, migrated cells were fixed with methanol (500 μL) and stained with 1% crystal violet solution (500 μL). The migration capacity of cells was observed by microscope (EVOS live cell imager system, Thermo Fisher, AMF7000) and photographed for counting. Cells were inoculated onto the chamber with Matrigel (Corning, New York, USA, 354,234) in the invasion experiment, and the rest of the procedures were the same as the migration experiment.

### Colony formation assay

100 μL cell suspension (5 × 10^3^ cells/mL) was added into the 6-well plate to culture for 2 weeks, and the medium was replaced with a fresh one every 3 days. After that, cells were fixed with 0.8 mL methanol for 20 min, and then stained with crystal violet (1%) for 15 min. The microscope was used for observation after being cleaned by PBS. Cell counting by Image J and the results were shown in a bar chart.

### Real-time reverse transcription PCR (qRT-PCR)

The total RNA was extracted from treated cells by Trizol reagent (Ambion, USA 15596018). Following reverse transcription using a cDNA synthesis kit (Takara, Kyoto, Japan, 6215B), qPCR analysis was performed on the ABI-7500 system (Thermo Fisher, 4,351,106). The relative expression levels of mRNAs were calculated utilizing the 2^−ΔΔCt^ method. The primers are shown in Table 1.

**Table 1:** Information of the primers

Names	F (5′−3′)	R (5′−3′)
G0S2	CGCAAGGGGAAGATGGTGAA	TGCTGCTTGCCTTTCTCCTG
ASS1	TCCGTGGTTCTGGCCTACA	GGCTTCCTCGAAGTCTTCCTT
COL12A1	AGCTGAGGCAGACATTGTGTT	CCTCCTTTGTACGGCAAGTTT
NCAPD2	TGGAGGGGTGAATCAGTATGT	GCGGGATACCACTTTTATCAGG
PLK1	AAAGAGATCCCGGAGGTCCTA	GGCTGCGGTGAATGGATATTTC
β-actin	AGCGGGAAATCGTGCGTG	CAGGGTACATGGTGGTGCC

### Western Blot (WB)

Protein was extracted from the treated cells with PIRA lysate, and then the protein concentration was determined with a BCA kit (Thermo Fisher, 23,235). The subsequent procedures performed followed the previous research (Zhang et al. 2024). The chemiluminescence kit (Thermo Fisher, USA, 20,148) was used to visualize the protein bands. β-actin served as a loading control. The information on antibodies is exhibited in Table 2.

**Table 2:** Information on the antibodies

Names	Brand	No.	Dilution rate
UTX	Abcam	ab300513	1:1000
E-cadherin	Santa Cruz	sc-8426	1:1000
N-cadherin	Santa Cruz	sc-59987	1:1000
Vimentin	Abclonal	A19607	1:2000
Ki67	Abcam	ab15580	1 μg/mL
G0S2	Abcam	Ab236113	1:1000
TLR4	Affinity	AF7017	1:1000
TRAF6	Abcam	Ab40675	1:5000
p-IKKα	CST	2697 T	1:1000
IKKα	CST	11930 T	1:1000
p-ERK	Proteintech	28,733–1-AP	1:1000
ERK	Proteintech	51,068–1-AP	1:1000
Bcl2	CST	4223 T	1:1000
Caspase3	CST	9662	1:1000
cleaved-caspase3	CST	9661 s	1:1000
cleaved-PARP	Proteintech	13,371–1-AP	1:1000
H3K27me3	Abcam	ab177178	1:10,000
H3K4me1	Abcam	ab8895	1:1000
H3	Proteintech	17,168–1-AP	1:2000
β-actin	CST	4970	1:5000

### Mice and tumor inoculation

In this study, the xenograft experiments were used to assess the role of UTX (both wide and mutant types) on PC tumor growth. The 54 male nude mice for 4 weeks were purchased from SLAC (Shanghai, China) and maintained in the designated environment (pathogen-free, day/night: 12/12, 22 ℃). 18 nude mice were prepared and randomly divided into 3 groups (n = 6). We subcutaneously injected stably UTX-Wt and UTX-Mut overexpressed PANC-1 cells (8 × 10^6^ cells/mice) and the corresponding control cells into the mice, respectively. The tumor growth was monitored and the mice were euthanized until the tumor volume reached 1500 mm^3^ (long diameter × short diameter^2^ × 1/2). The tumor was stripped and volume was measured with a caliper (SYNTEK, Hangzhou, SY-119–200). The weight of the tumor was also measured.

Additionally, for further detecting the role of UTX in PC drug resistance in vivo, another xenograft experiment was performed (36 nude mice, n = 6). Gemcitabine (intraperitoneal injection, 60 mg/kg, 2 times per week for 6 weeks) was used to treat the mice with PANC-1 cells in normal control, UTX overexpressed (both wild and mutant type), and UTX-Mut + G0S2 (8 × 10^6^ cells/mice) overexpressed groups. Chloroquine (oral gavage, 10 mg/kg, 5 times per week for 4 weeks) combined with gemcitabine was used to treat mice with UTX-Mut overexpressed PANC-1 cells. DMSO (intraperitoneal injection, 2 times per week for 6 weeks) in the same volume as gemcitabine was treated mice with PANC-1 cells as a control group. The rest of the steps were the same as the above experiment. All procedures performed in this research followed the protocol of the Animal Ethics Committee of Jiangxi Cancer Hospital (the approval number: 2021ky034).

### TUNEL assay

TUNEL assay was used to detect cell apoptosis in xenograft tissues. Tumor tissues were embedded in paraffin wax, sectioned with a thickness of 5 μm. The slices were paraffinized in xylene, hydrated in ethanol on different gradients. Then 100 μL proteinase K solution (20 μg/mL) was added for permeability. According to the instructions of the TUNEL kit (Servicebio, Wuhan, G1501-50 T), the FITC solution was incubated at 37 ℃ for 1 h without light, then washed with PBS. After nuclear staining with DAPI solution, the sections were sealed, photographed under a fluorescence microscope immediately.

### Immunohistochemistry (IHC)

Tumor tissue slices were treated with citrate buffer (Servicebio, Wuhan, China, G1202) and 3% H_2_O_2_, respectively. Then, the slices were blocked with 3% BSA, the primary antibody was incubated at 4 ℃ overnight, and the secondary antibody was incubated at room temperature for 30 min. The staining and re-staining were completed with DAB (Servicebio, G1212) and hematoxylin (Servicebio, G1004), respectively. Finally, the pictures were taken under the microscope.

### Bioinformatics

The total RNA of UTX-Wt and UTX-Mut overexpressed PANC-1 cells, and their corresponding control were collected for mRNA sequencing by LC-BIO (Hangzhou, China). The data was analyzed by DAVID software (https://david.ncifcrf.gov/) to gain differentially expressed genes (DEGs), and analysis of heatmaps and volcanic by the R program. Kyoto Genome Encyclopedia (KEGG) and Gene Ontology (GO) were used to analyze the enriched signaling pathways of DEGs.

### Flow cytometry

The cell apoptosis was detected by flow cytometry. The treated cells were re-suspended in 500 μL PBS, followed by staining of Annexin V-FITC and PI solution according to the instruction of Annexin V-FITC apoptosis kit (Beyotime, Shanghai, C1062S), respectively. After incubation, flow cytometry (Thermo Fisher) was used for apoptosis detection immediately.

### Chromatin immunoprecipitation (CHIP)

According to the instruction of the CHIP-Seq High Sensitivity Kit (Abcam, Cambridge, UK, ab185908), PANC-1 cells overexpressing UTX were cross-linked, cleaved, and then ultrasound at 4 ℃ to obtain the fragmented DNA. Subsequently, immunoprecipitation was performed using antibody and protein A/G MagPoly Beads. Then DNA Clean-Up Kit (Omega, Norcross, GA, USA, D6296-01) was used to purify DNA, and the enrichment degree of target gene promoter was validated by qRT-PCR. The primer sequence of the G0S2 1–6 promoter is as follows (see Table 3):

**Table 3:** Information of G0S2 promoter primers

Names	F (5′−3′)	R (5′−3′)
Primer-1	TCCGACCGACTGCAAAGG	GGCTGAGGGAGCGTTCTA
Primer-2	AAAGCCAAACTAGAACGC	TCACTGCTCCAGCTCAAC
Primer-3	AAAGCACCATCAATGAATAG	GGACAGAACAGGTTACGC
Primer-4	AAAGCACCATCAATGAATAG	GGACAGAACAGGTTACGC
Primer-5	GTGACAACCCTTCCGAATAAA	CTCTGACATGGCGGCAAC
Primer-6	GGTGGTGGGAGCCGTTCTT	CGCTTCTGGGCCATCATCT

### Statistical analysis

The results came from at least three repetitive experiments. All representative data was analyzed by GraphPad Prism 6.0 and presented as the mean ± standard deviation (SD). Statistical analysis was performed utilizing a t-test for two-group comparison and one-way ANOVA for multiple-group comparison. **P < 0.01, *P < 0.05 were deemed significant.

## Results

### Mutation on the jmjC domain of UTX effectively impaired its antitumor effect in PC cells.

**Fig. 1:**
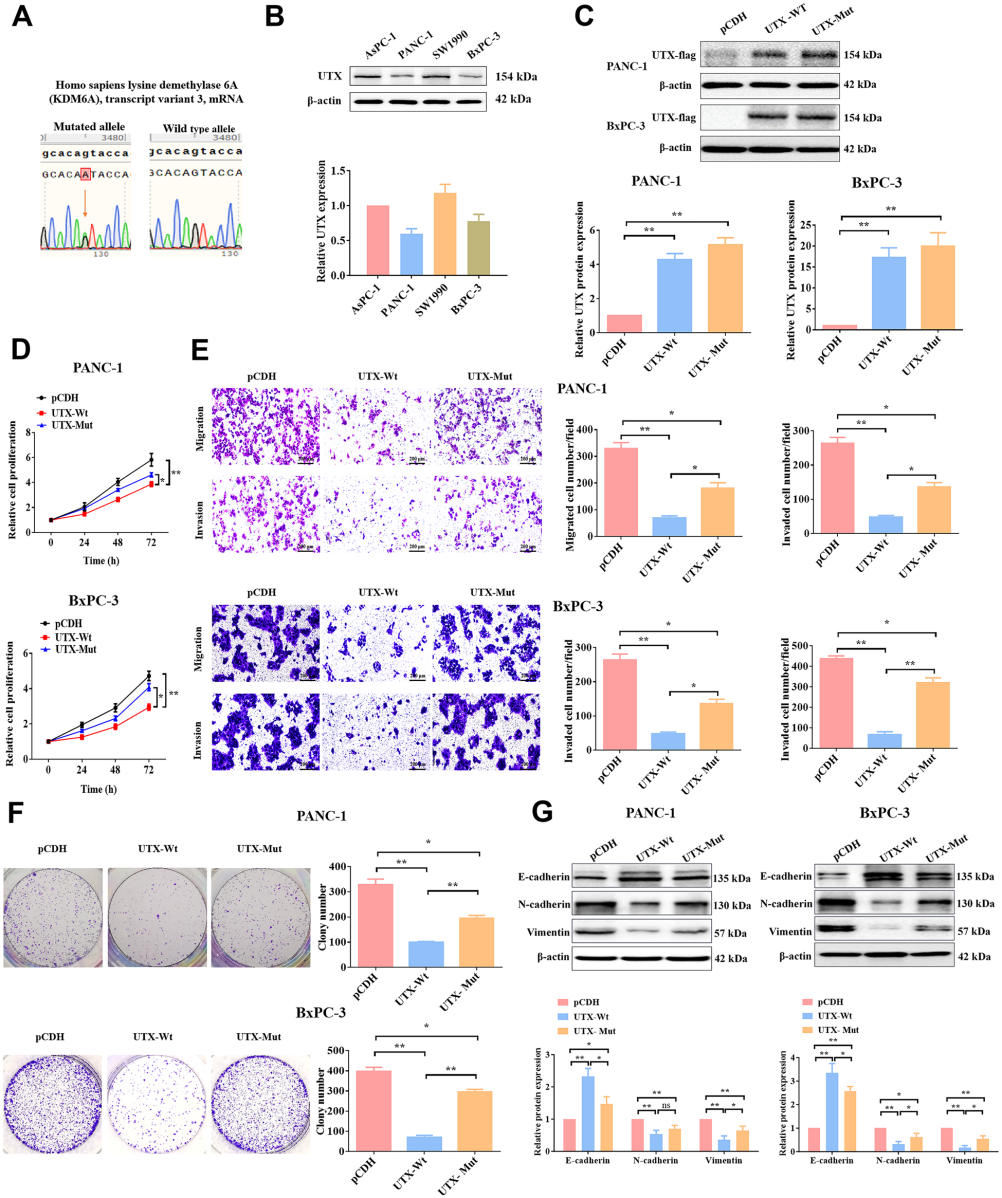
Mutant UTX reduced wild-type UTX’s inhibition of malignant behavior in PC cells. **A** The exome sequencing analysis of PC samples. **B** The expression levels of UTX in the four PC cells (AsPC-1, PANC-1, SW1990, and BxPC-3) were detected by WB. **C** The WB assay was performed to verify the overexpressed efficiency of mutant UTX and wild-type UTX in PANC-1 and BxPC-3 cells. The abilities of proliferation (**D**), migration, invasion (**E**), and colony formation (**F**) were examined by MTT, transwell, and colony formation assays, respectively. Scale: 200 μm. **G** The expression of EMT-related proteins was also examined by WB assay. All data came from at least three repeated experiments. *P < 0.05, **P < 0.01

It has been reported that the UTX gene encodes the histone H3K27 demethylase, acting as a tumor suppressor, which is frequently mutated in human cancers (Wang et al. 2012). In this study, the result of exome sequencing revealed that there was a missense mutation (G → A) on the exon 21 of UTX’s jmjC domain in PC (Fig. 1A). Sequencing of 12 PC samples showed that this missense mutation rate on exon 21 was 75% (9/12). We examined UTX expression in four PC cell lines, and PANC-1 and BxPC-3 showed low expression (Fig. 1B). To understand the role of this missense mutation of UTX on the progression of PC, we selected these cells for experiments to explore its impact on cellular functions. First, we constructed UTX overexpressed PANC-1 or Bxpc-3 cell lines (the wild type one and the mutant one, respectively), and WB experiments were performed to verify the overexpression efficiency (Fig. 1C). Interestingly, the wild-type UTX significantly inhibited cell proliferation, migration, invasion, and clonogenic abilities (Fig. 1D–F), which was consistent with the previous reports (Nickerson et al. 2014). Conversely, these phenotypes were reversed in the UTX-mutated PC cells. For further exploration, we examined the expression levels of crucial protein biomarkers associated with epithelial-mesenchymal transition (EMT), a process crucial for promoting cell motility and tumor metastasis (Xu et al. 2023). Not surprisingly, overexpression of UTX-Wt led to increased expression of E-cadherin, accompanied by decreased expression of N-cadherin and Vimentin in PC cells, whereas these effects were notably reversed by UTX-Mut overexpression (Fig. 1G).

**Fig. 2:**
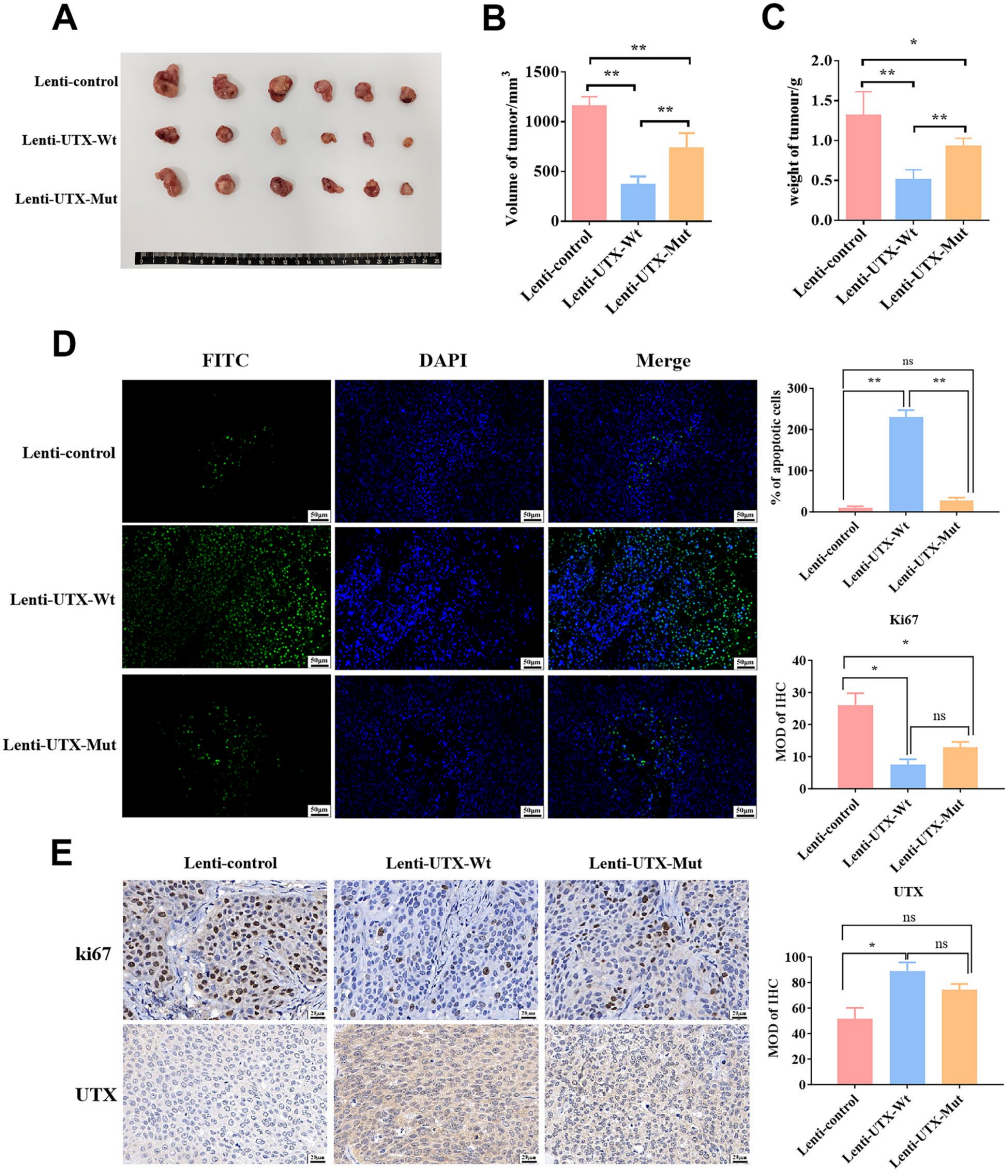
Mutant UTX enhanced PC malignancy more than wild-type UTX in vivo. The nude mice (n = 6) were subcutaneously injected with stably wild-type UTX or mutant UTX overexpressed PANC-1 cells (8 × 10^6^ cells/mice), respectively. **A** The tumor tissues were stripped after the euthanasia of mice. (**B**, **C**) The weight (**B**) and volume (C) of tumor tissues were measured. The rate of apoptosis (**D**) was examined by TUNEL assay (Scale: 50 μm), and the expression of ki67 and UTX (**E**) in tumor tissues was examined by IHC assay (Scale: 20 μm), respectively. All data came from at least three repeated experiments. *P < 0.05, **P < 0.01

Subsequently, the xenograft models were constructed to validate the role of the mutated UTX in vivo. As anticipated, the overexpressed UTX-Wt notably inhibited the growth of xenograft tumors, which were significantly accelerated in the UTX-Mut group (Fig. 2A–C). Consistently, the UTX-Mut significantly impaired the pro-apoptosis effect of UTX-Wt, and the expression of Ki67 and UTX was higher in the tumor tissues with UTX mutated compared to those overexpressing wild-type UTX (Fig. 2D, E). Taken together, our results preliminarily confirmed that this missense mutation on the JmjC domain of UTX contributed to impair its anti-tumor effect in PC.

### UTX mutation weakened the expression of G0S2 and activated the Toll-like signaling pathway

**Fig. 3:**
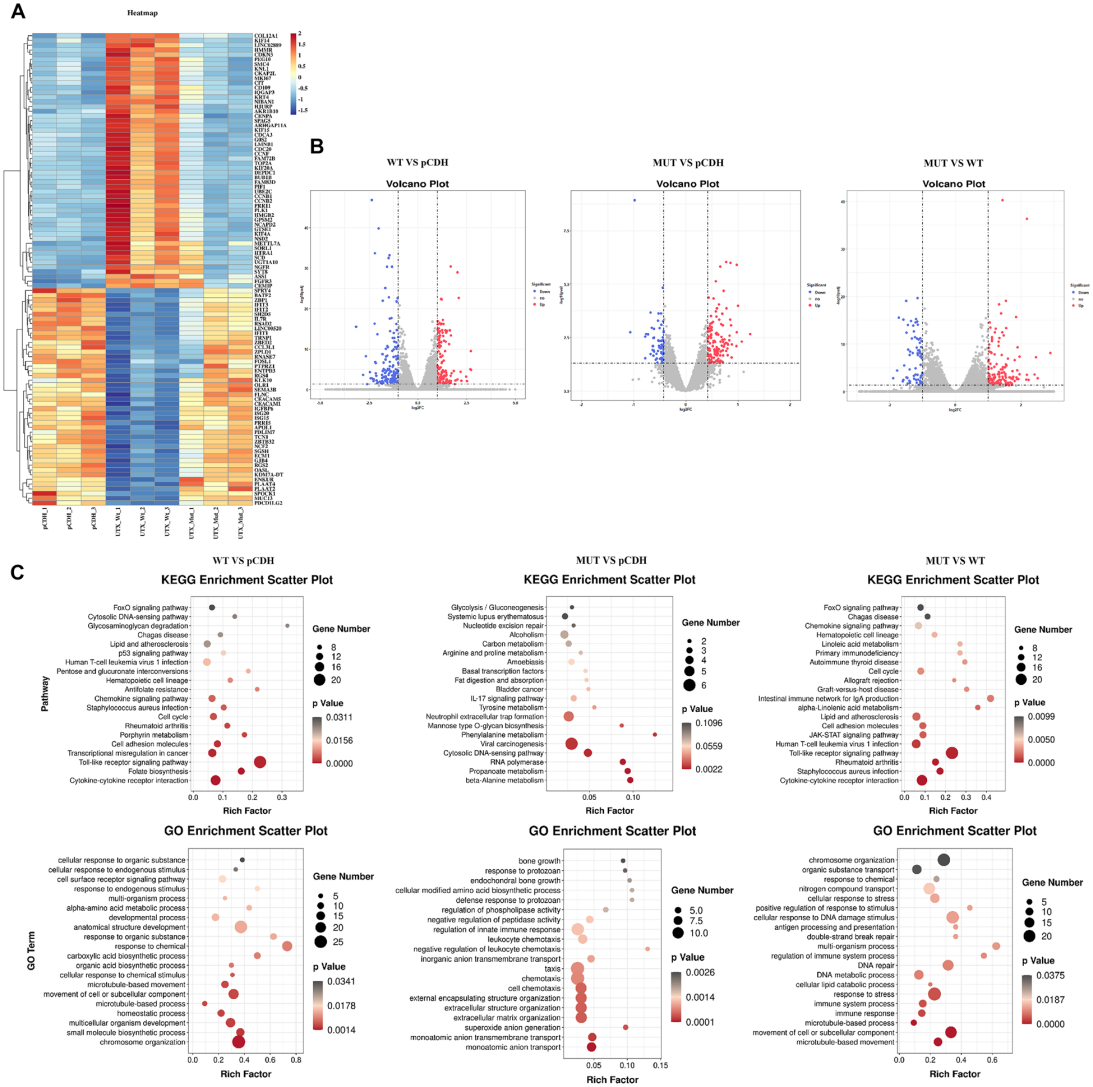
DEGs were analyzed after overexpression of wild-type UTX and mutant UTX in PANC-1 cells. The total RNA was extracted after overexpression of wild-type UTX and mutant UTX in PANC-1 cells and DEGs were analyzed using the mRNA-seq. The heatmap (**A**), volcanic (**B**), GO enrichment analysis, and KEGG enrichment analysis (**C**) of wild-type UTX vs pCDH (control), mutant UTX vs pCDH (control), and mutant UTX vs wild-type UTX

Next, we attempted to explore the mechanism by which UTX mutation promoted the PC progression. As shown in Fig. 3A, B, a total of 293 DEGs were identified from the sequencing data comparing the wild-type UTX group with the pCDH group (control group), with 137 genes upregulated and 156 genes downregulated. Furthermore, upon UTX mutation, 150 genes were up-regulated and 74 genes were down-regulated compared to the wild-type UTX group. The outcomes derived from GO and KEGG enrichment analyses showed that these DEGs were mainly involved in cell adhesion, chemical synaptic transmission, multicellular signal transduction, and the Toll-like receptors signaling pathway (Fig. 3C).

**Fig. 4:**
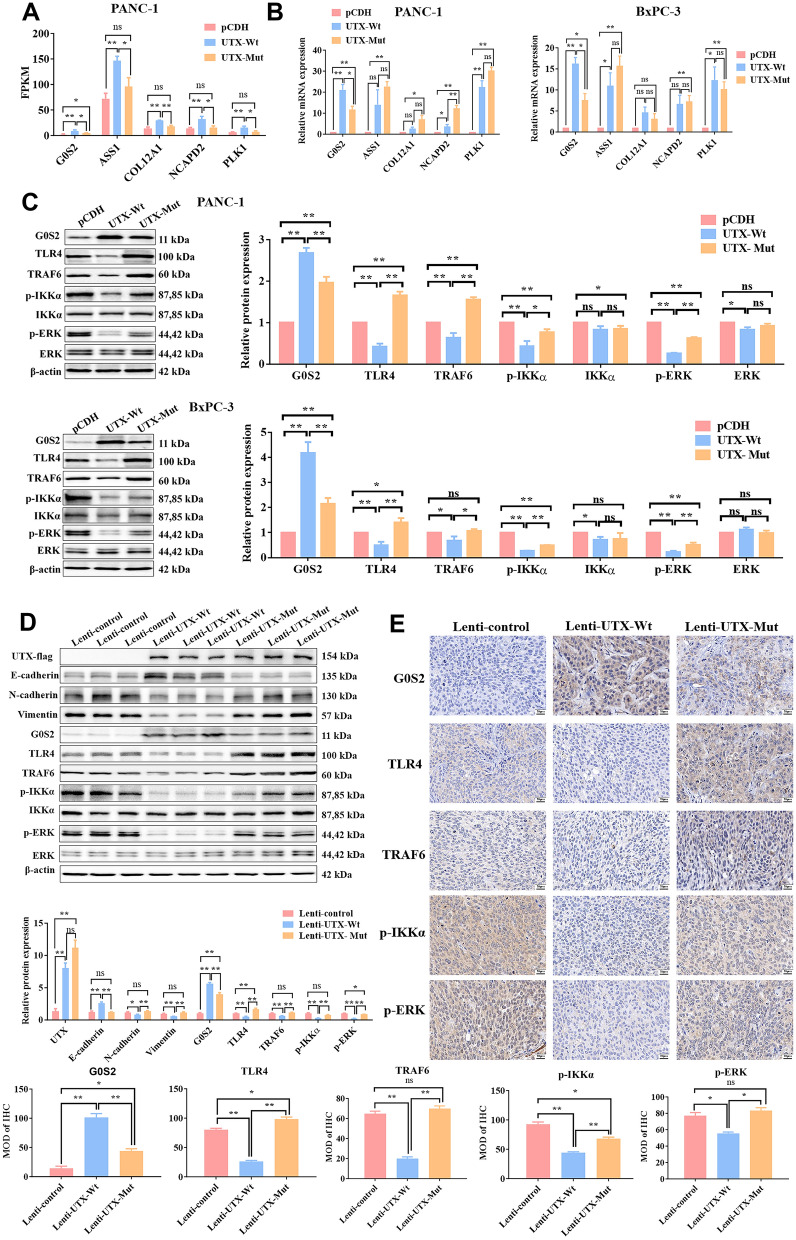
Mutant UTX, via G0S2/Toll-like pathways, accelerates PC progression more than wild-type. **A**, **B** The mRNA expression of DEGs by sequencing analysis (**A**) and PCR assay (**B**). **C**, **D** The protein levels of G0S2 and Toll-like-related factors were examined by WB assay in vitro (**C**) and in vivo (**D**). **E** The expression of G0S2 and Toll-like-related factors was examined by IHC in *vivo*. Scale: 20 μm. All data came from at least three repeated experiments. *P < 0.05, **P < 0.01

Fragments Per Kilobase Million (FPKM) is an indicator derived from RNA-Seq technology, estimating gene expression by considering both the quantity and length of detected RNA sequences (Zhao et al. 2021). Through further FPKM and qRT-PCR verification, the significant change in G0S2 expression was worthy of our attention, since only G0S2 consistently showed differences between the two assays. The result revealed that the expression of the G0S2 was upregulated in wild-type UTX PC cells, which was attenuated by the mutation of UTX (Fig. 4A, B). Substantial evidence has indicated that G0S2 functions as a tumor suppressor by repressing PI3K/Akt/mTOR activity which is associated with Toll-like/MAPK signaling pathways (Yim et al. 2017; Gajanayaka et al. 2021). Our KEGG analysis showed DEGs enriched in the Toll-like receptor pathway, thus, we examined its related proteins expression, including Toll-like Receptor 4 (TLR4), TNF Receptor-Associated Factor 6 (TRAF6), Phosphorylated IκB Kinase α (p-IKKα), and Phosphorylated Extracellular Signal-Regulated Kinase (p-ERK), to assess UTX’s effect on Toll-like signaling in PC cells. In our endeavor, we found that the expression of G0S2 was significantly increased and the expression of biomarkers of Toll-like signaling pathway (TLR4, TRAF6, p-IKKα, and p-ERK) was markedly decreased in PANC-1 and BxPC-3 expressed wild-type UTX, which were attenuated by UTX mutation (Fig. 4C). More importantly, these results were further validated in vivo (Fig. 4D, E).

### G0S2 Overexpression inhibited the tumor malignant phenotype and the drug resistance for Gemcitabine in PC cells

**Fig. 5:**
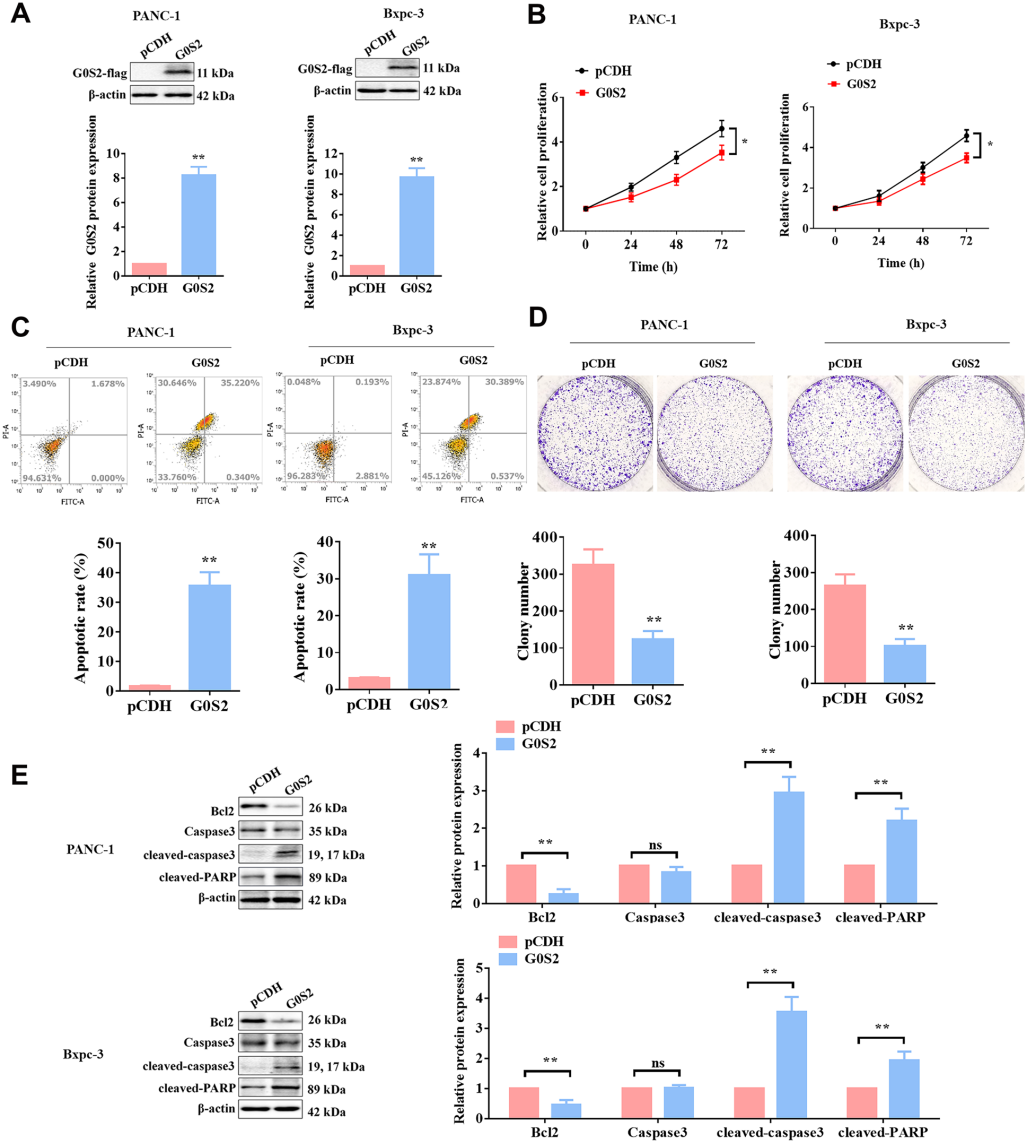
G0S2 overexpression inhibited the malignant phenotype of PC cell lines. **A** Construction of G0S2 overexpression in PANC-1 and BxPC-3 cells by pCDH vector and WB assay was performed to validate the efficiency of overexpressed G0S2 in PANC-1 and BxPC-3 cells. **B**–**D** After overexpression of G0S2 in PANC-1 and BxPC-3 cells, the abilities of proliferation (**B**), apoptosis (**C**), and clone formation (**D**) were examined by MTT, flow cytometry, and clone assays, respectively. **E** The expression of apoptosis-related proteins was examined by WB assay. All data came from at least three repeated experiments. *P < 0.05, **P < 0.01

Numerous studies have identified the specific interaction between G0S2 and Bcl-2 at the mitochondria, disrupting the formation of the Bcl-2/Bax anti-apoptotic heterodimer complex, thereby regulating its anti-apoptotic activity in human tumor cells (Heckmann et al. 2013; Wang et al. 2015). Furthermore, the loss of G0S2 promotes disease progression and drug resistance in chronic myeloid leukemia (CML) by disrupting glycerophospholipid metabolism (Gonzalez et al. 2022). To further determine the role of G0S2 in PC progression, the G0S2-overexpressed PANC-1 or Bxpc-3 cell lines were constructed respectively (Fig. 5A). As shown in Fig. 5B–D, the overexpression of G0S2 inhibited cell proliferation and clonogenic abilities, while promoting the cell apoptosis. Consistently, changes in the expression of proteins associated with apoptosis showed the down-regulation of Bcl2 and the up-regulation of Caspase3, cleaved-caspase3, and cleaved-PARP (Fig. 5E). Additionally, we also constructed a G0S2-silenced PANC-1 cell line and observed that the knockdown of G0S2 in wild-type UTX overexpression PC cells would reverse the tumor suppressor function of UTX, as shown in Figure S1.

**Fig. 6:**
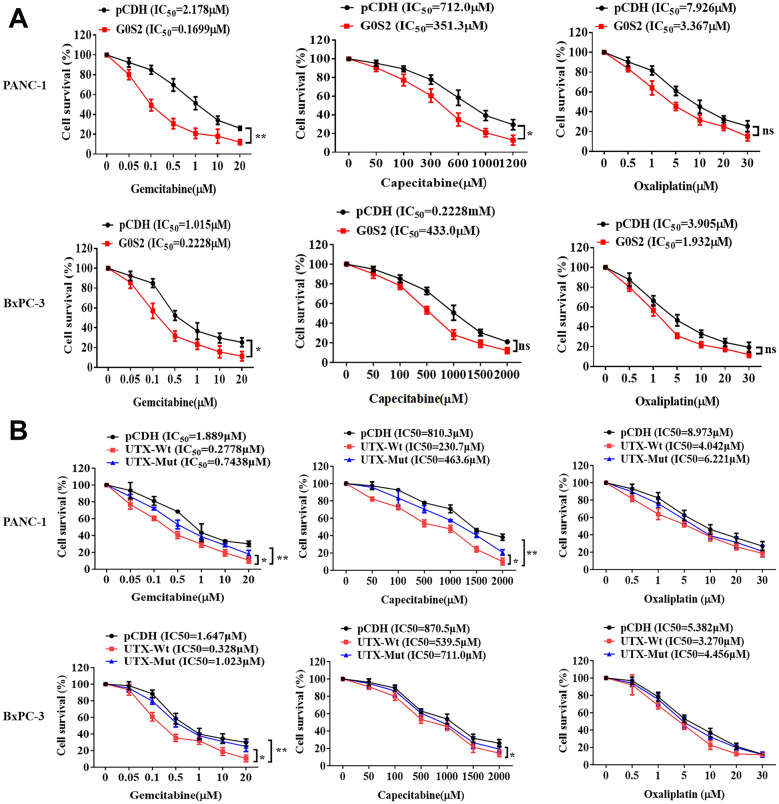
The drug resistances for Gemcitabine, Capecitabin, and Oxaliplatin in PANC-1 and BxPC-3 cells. **A** The drug resistances for Gemcitabine, Capecitabine, and Oxaliplatin were examined in PC cells with or without overexpression of G0S2 by MTT assay. **B** The drug resistances for Gemcitabine, Capecitabine, and Oxaliplatin were examined in PC cells with wild-type UTX or mutated UTX by MTT assay. All data came from at least three repeated experiments. *P < 0.05, **P < 0.01

**Fig. 7:**
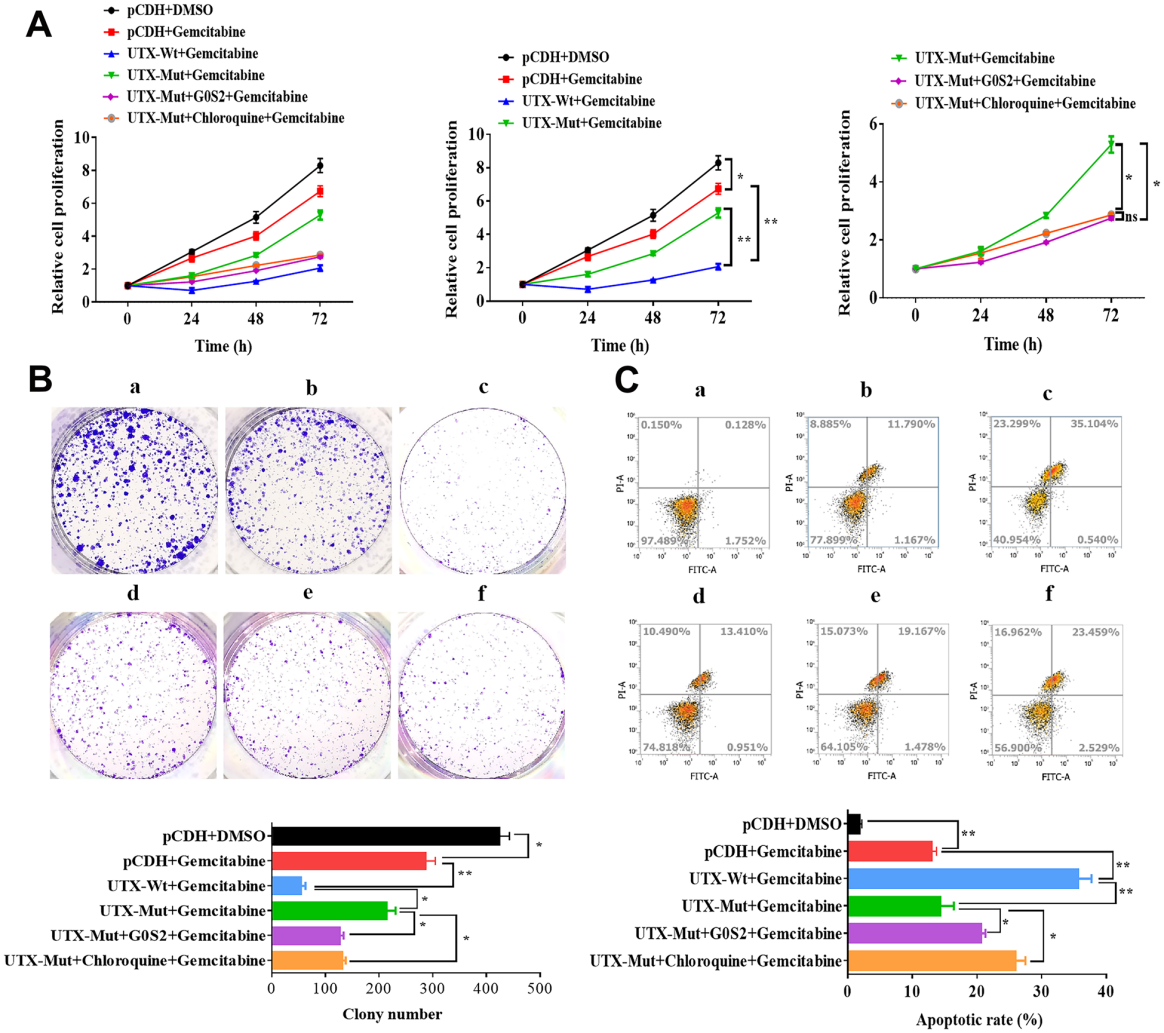
G0S2/Toll-like pathways overexpression curbs PANC-1 malignancy and Gemcitabine resistance. The PANC-1cell line was given Gemcitabine (100 nM, 24 h) along with different treatments. The groups of different treatment follow as (a) pCDH + DMSO, (b) pCDH + Gemcitabine, (c) UTX-Wt + Gemcitabine, (d) UTX-Mut + Gemcitabine, (e) UTX-Mut + G0S2 + Gemcitabine, (f) UTX-Mut + Chloroquine (2 μM, 24 h) + Gemcitabine. **A**–**C** The proliferation (**A**), clone formation ability (**B**), and cell apoptosis (**C**) were examined by MTT, clone formation, and flow cytometry assays, respectively. All data came from at least three repeated experiments. *P < 0.05, **P < 0.01

**Fig. 8:**
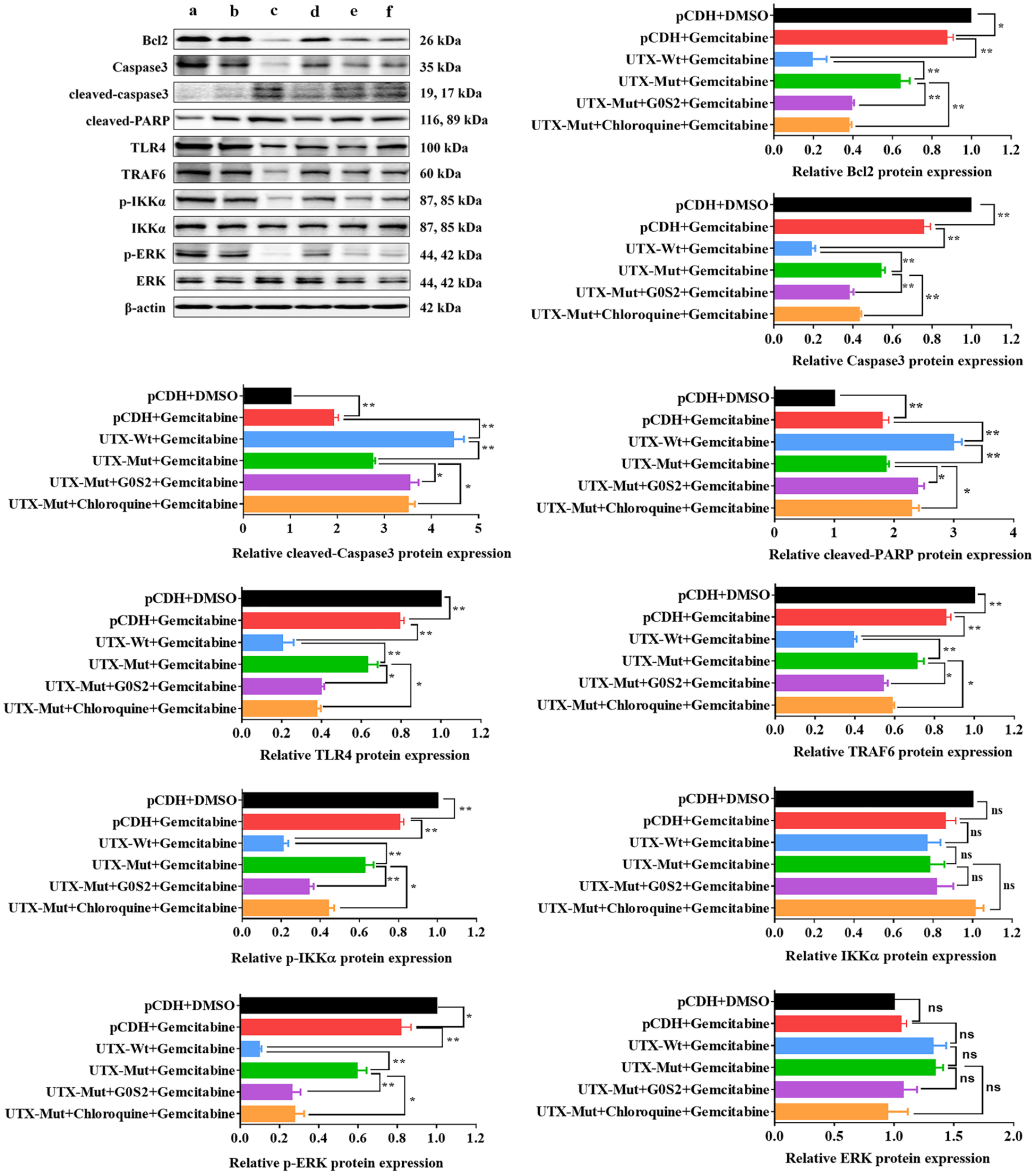
Gemcitabine resistance in PANC-1 cells curbed by Toll-like/G0S2 pathways and G0S2 overexpression. The PANC-1cell line was given Gemcitabine (100 nM, 24 h) along with different treatments. The groups of different treatment follow as (**a**) pCDH + DMSO, (**b**) pCDH + Gemcitabine, (**c**) UTX-Wt + Gemcitabine, (**d**) UTX-Mut + Gemcitabine, **(e**) UTX-Mut + G0S2 + Gemcitabine, (**f**) UTX-Mut + Chloroquine (2 μM, 24 h) + Gemcitabine. **A** The expression levels of apoptosis-related proteins and biomarkers of Toll-like signaling pathway were examined by WB assay. All data came from at least three repeated experiments. *P < 0.05, **P < 0.01

**Fig. 9:**
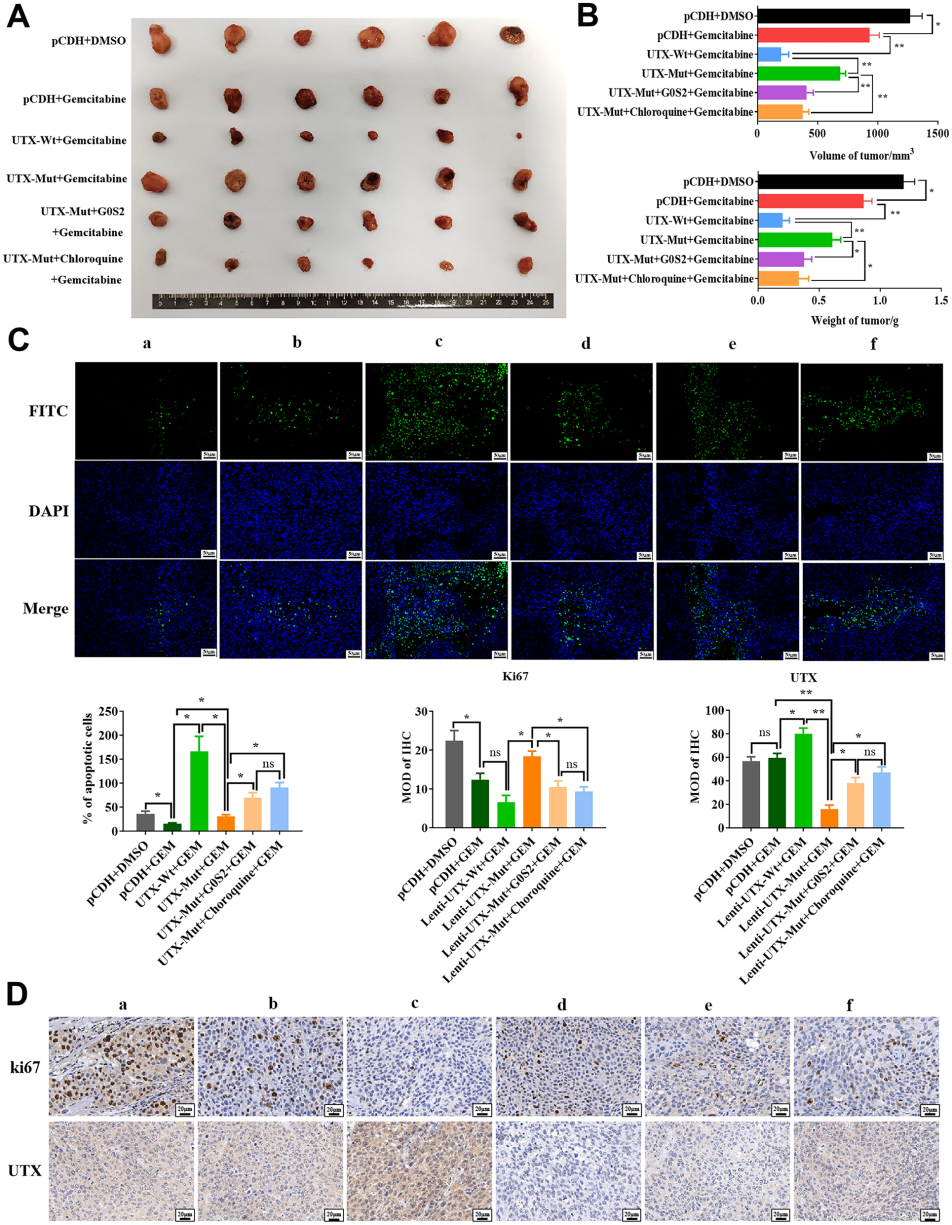
Gemcitabine resistance in PC in* vivo* curbed by Toll-like/G0S2 pathways and G0S2 overexpression. The nude mice were divided into 6 groups (n = 6). Gemcitabine (60 mg/kg, intraperitoneal injection, 2 times per week for 6 weeks) and chloroquine (10 mg/kg, oral gavage, 5 times per week for 4 weeks) were treated to mice which were subcutaneously injected with stably UTX-Wt, UTX-Mut, and G0S2 overexpressed in PANC-1 cells (8 × 10^6^ cells/mice), respectively. The control group was subcutaneously injected with DMSO for 6 weeks. **A** The tumor tissues were stripped after the euthanasia of mice. **B** The weight and volume of tumor tissues were measured. The rate of apoptosis **C** was examined by TUNEL assay (Scale: 50 μm), and the expression of ki67 (**D**) in tumor tissues was examined by IHC assay (Scale: 20 μm), respectively. The group information of (a–f) was consistent with Fig. 8A. All data came from at least three repeated experiments. *P < 0.05, **P < 0.01

Subsequently, we wondered whether UTX mutation-induced changes in G0S2 expression was involved in the drug resistance of PC. After being treated with Gemcitabine, Capecitabine, and Oxaliplatin, the cell survival of PANC-1 and BxPC-3 cells with or without overexpression of G0S2 was assessed. The results showed that overexpression of G0S2 reduced drug IC50 in PC cells, with significant decreases for Capecitabine, Oxaliplatin (~ 50%), and Gemcitabine (Fig. 6A). UTX mutation reduced this effect, especially for Gemcitabine (Fig. 6B). These results indicated that UTX mutation-induced G0S2 overexpression could inhibit drug resistance in PC cells. We further explored whether UTX-regulated G0S2 and Toll-like signaling pathways affect Gemcitabine’s anti-tumor effect on PC cells. As shown in Fig. 7A (To facilitate understanding and analysis of the results, the first graph was split into two statistical graphs on the right), overexpression of wild-type UTX exhibited the highest inhibitory effect of Gemcitabine on proliferation of PC cells. UTX mutation weakened this effect, but G0S2 overexpression or Chloroquine (a Toll-like pathway inhibitor) treatment could attenuate the resistance to Gemcitabine caused by UTX mutation in PANC-1 cells. Consistently, these results were also observed in colony formation and apoptosis of PC cells (Fig. 7B, C). More importantly, the impairing effect of the mutated UTX on Gemcitabine could be effectively reversed by G0S2 overexpression or the application of the Toll-like inhibitor, as corroborated in the associated protein expression levels (Fig. 8A). Convincingly, this conclusion was also confirmed by the in vivo experiments (Fig. 9A–D). Taken together, these results demonstrated that the overexpression of G0S2 effectively suppressed the tumor malignant phenotype in PC and promoted the sensitization of PC cells to Gemcitabine.

### UTX up-regulated G0S2 expression by inhibiting its H3K27me3 modification

**Fig. 10:**
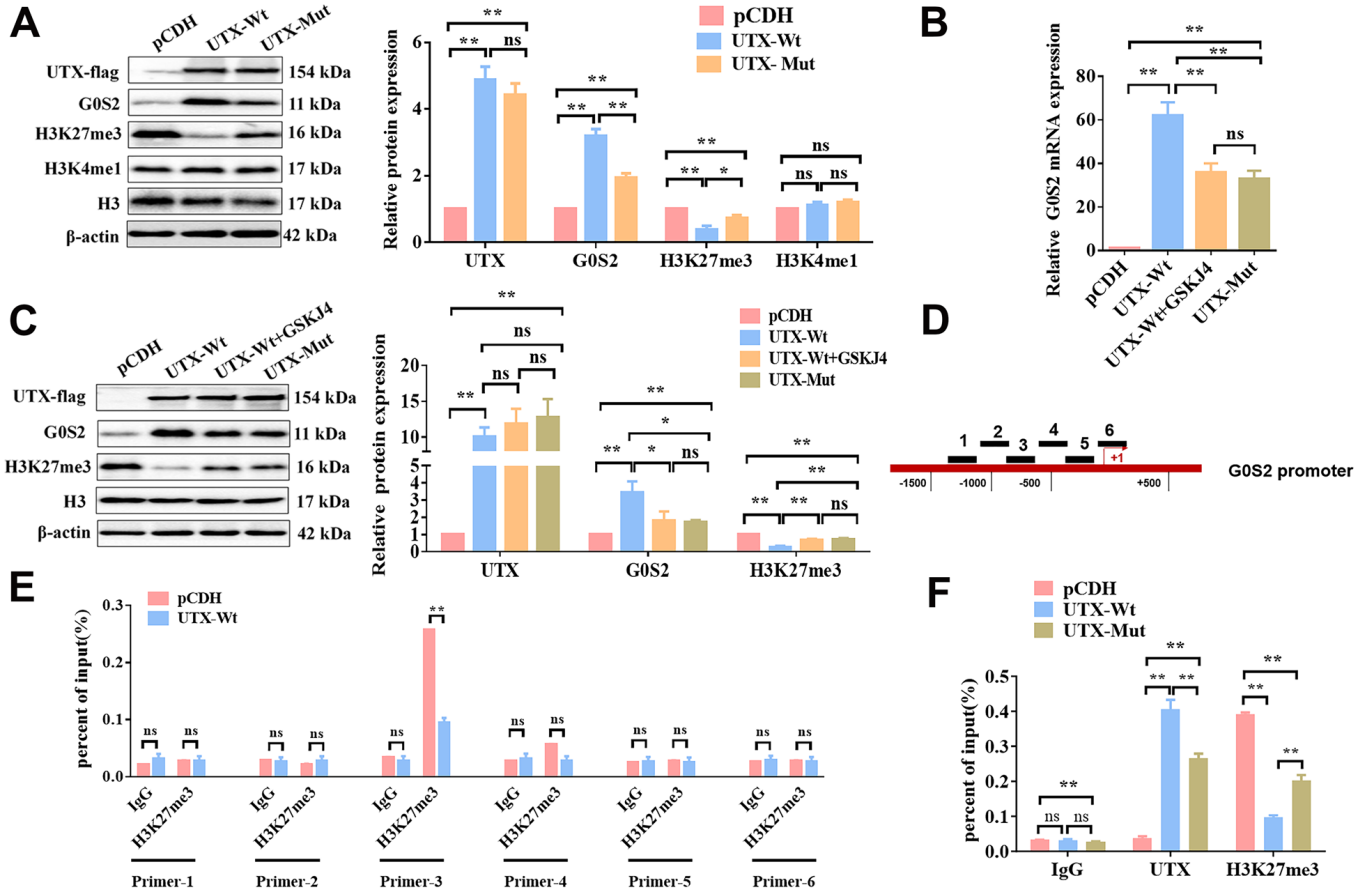
UTX promoted the expression of G0S2 by reducing the H3K27me3 modification level of its promoter. **A** WB assay was used to examine the expression of UTX-flag, G0S2, H3K27me3, H3K4me1, and H3 in PANC-1 cells with pCDH, UTX-Wt, or UTX-Mut, respectively. **B** The mRNA levels of G0S2 were examined by qRT-PCR in PANC-1 cells with pCDH, UTX-Wt, UTX-Wt + GSKJ4, or UTX-Mut, respectively. **C** The protein levels of UTX-flag, G0S2, and H3K27me3 were examined by WB assay. **D** The promoter fragment of G0S2. CHIP and qRT-PCR assays were used to examine the H3K27me3 modification level of the G0S2 promoter 1–6. **E** The expression of UTX and **F** H3K27me3 modification levels in PANC-1 cells with pCDH, UTX-Wt, or UTX-Mut, respectively. All data came from at least three repeated experiments. *P < 0.05, **P < 0.01

It has been well documented that UTX participates in H3K27me3 demethylation and then activates gene expression, and GSKJ4 can effectively inhibit the activity of H3K27me3 histone demethylase (Yan et al. 2017). As such, we speculated that the G0S2 expression was regulated by UTX-mediated H3K27me3 modification. Interestingly, the expression of G0S2was increased and the H3K27me3 level was decreased in PC cells with wild-type UTX overexpressed. Conversely, these effects were reversed in PC cells with the mutated UTX overexpressed, and moreover, the treatment of GSKJ4 (a small molecule inhibitor that specifically targets the histone demethylase enzyme known as UTX) showed a similar effect as UTX mutation in regulating the G0S2 and H3K27me3 levels (Fig. 10A, B, C). Not surprisingly, the subsequent CHIP experiments confirmed that there was a binding relationship between UTX and G0S2 promoter-3 via H3K27me3 modification (Fig. 10D, E). More importantly, our data indicated that wild-type UTX exhibited a higher enrichment on the G0S2 promoter than mutated UTX, with a lower level of H3K27me3 modification (Fig. 10F). Taken together, we concluded that wild-type UTX activated the expression of G0S2 through down-regulating H3K27me3 modification, which can be significantly attenuated by the missense mutation on the jmjC domain of UTX.

## Discussion

As an important part of the post-translational modification of histone tails, histone lysine methylation was deeply involved in chromatin organization and gene expression (Lu et al. 2012). UTX is a histone demethylase located in the X chromosome (Zhang et al. 2024). It has been well documented that the JmjC domain of UTX can specifically target H3K27me3 histone for demethylation to activate the target gene expression (Shi et al. 2021). However, the mutations of UTX, mainly in the functional region of the JmjC domain, are frequently associated with various cancers (Ntziachristos et al. 2014; Ezponda et al. 2017; Wang et al. 2019). Gillian L. Dalgliesh et al. reported that overlap in transcriptional deregulation caused by UTX loss was one of the reasons identified as ccRCC cancer (Dalgliesh et al. 2010). Wu et al. discovered that loss of UTX resulted in elevated H3K27me3 levels to partially increase EZH2 expression in lung tumorigenesis (Wu et al. 2018). In bladder cancer, approximately 70% of UTX mutations resulted in a complete loss of expression and thus demethylase function (Barrows et al. 2020). Up to now, little is known about whether UTX is also mutated in PC. In our previous study, we formally demonstrated the anti-oncogenic activity of UTX in PC. In this study, we tried to identify the mutation of UTX in PC, and to further elucidate the underlying mechanisms by which the mutant UTX functioned.

By exon sequencing in PC tissues, we unexpectedly observed a missense mutation on the JmjC domain of the UTX. Therefore, an overexpression plasmid for this mutant UTX was constructed to test its roles in PC. Of note, in our study, it was found that wild-type UTX significantly inhibited EMT and suppressed the malignant phenotypes of PC cells, which could be effectively weakened by the mutant one. More importantly, this missense mutation of UTX significantly promoted the growth and inhibited apoptosis of xenograft tumors derived from PC cells, in comparison to the wild-type one. Therefore, our data indicated that this missense mutation on the JmjC domain of UTX may play a critical role in the regulation of pancreatic cancer development and progression.

Next, we tried to elucidate the mechanism by which this mutant UTX regulated the malignant phenotypes of PC cells. In this study, mRNA sequencing was used to screen the target genes of UTX, and our data linked the function of mutant UTX with the Toll-like signaling pathway and the G0S2 gene. Numerous studies have reported that Toll-like receptors sense pathogen-associated molecular patterns (eg: lipopolysaccharides), and then several kinases and ubiquitin ligases (TRAF6 and pellino 1) are activated, resulting in the induction of the p38 MAP kinase (MAPK), NF-B, type I interferon, and JNK MAPK pathways (Hennessy et al. 2010; Kawai and Akira 2010). Arum Kim and Chung Soo Lee believed that apigenin has the potential to suppress the lipopolysaccharide-caused inflammatory mediator production in keratinocytes by reducing the activation of JNK and p38-MAPK, as well as the Toll-like receptor-4-dependent activation of Akt, mTOR, and NF-κB pathways (Kim and Lee 2018). At the same time, Christina et al. revealed that G0S2 acts as a tumor suppressor in breast cancer by regulating PI3K/mTOR activity, and G0S2 improves therapeutic responses to PI3K/mTOR inhibitors (Yim et al. 2017). G0S2 was originally discovered to be transiently activated in lymphocytes during the G0 to G1 transition (Wang et al. 2019). Numerous studies have indicated that G0S2 regulates energy consumption, lipid metabolism, and immunological function through unique protein–protein interactions in many tissues (Kioka et al. 2014; Nielsen and Moller 2014). In NSCLC, there was an inverse correlation between G0S2 mRNA expression and DNA methylation, and it was reported that G0S2 was fully methylated and inhibited in LC-1 sq, one of the squamous lung cancer cell lines (Kusakabe et al. 2009, 2010). Recent research indicated that G0S2 decreased cell proliferation by inhibiting the catalyzed lipolysis of adipose triglyceride lipase (ATGL) (Yang et al. 2010). Strikingly, in our study, we found that G0S2 was down-regulated, and the key markers Toll-like were up-regulated in the UTX mutation group compared with the wild-type UTX group in PC cells. More importantly, our results further verified that the malignant phenotype of pancreatic cancer was inhibited by overexpressed G0S2. The G0S2 gene has recently been identified as a target gene for all-trans retinoic acid and peroxisome proliferator-activated receptor (PPAR) agonists (Ma et al. 2013; Sun et al. 2018). Christina Y. Yim et al. also confirmed that G0S2 suppressed breast cancer by repressing a Myc-regulated transcriptional program which was known to favor tamoxifen resistance (Yim et al. 2016). The mystery of G0S2’s role in pancreatic cancer has yet to be solved. At present, only one study on the bioinformatics analysis of 179 pancreatic cancer samples and 171 normal pancreatic tissue samples on the GEPIA platform revealed that G0S2 was identified as a lipid drop-related factor and was differentially expressed in PC samples (Bai et al. 2021). Here, we found that wild-type UTX could significantly reduce Gemcitabine resistance in PC cells, which could be impaired by mutant UTX via down-regulating the expression of G0S2 and inactivating Toll-like signaling pathways. The most important is that this function was further validated in the xenograft experiments. To sum up, we determined that UTX might act as a tumor suppressor in PC by promoting the expression of G0S2, while the mutant UTX partially loses this function.

Finally, we further elucidated how the mutation of UTX affects its role in the regulation of G0S2 expression in PC cells. Our data revealed that the mutation of UTX indeed impaired its H3K27me2/me3 demethylation activity, as well as its promotion effect on G0S2 expression. However, it is unknown whether the expression of G0S2 in cancer cells has been involved in H3K27me3 up to now. The data from CHIP experiments further clarified that UTX indeed decreased the level of H3K27me3 modification on the promoter of the G0S2 gene, while the mutation of UTX significantly reversed this function. Meanwhile, the results from the CHIP assay also proved that UTX could directly bind to the promoter of the G0S2 gene, and the mutant UTX lost this function. In our endeavor, we confirmed that UTX promoted the expression of G0S2 by reducing the H3K27me3 modification level of its promoter, while this function could be significantly impaired by mutation on its JmjC domain in pancreatic cancer cells. However, there are some limitations in this study. First, the missense mutation of UTX in PC is not present in all samples, suggesting non-universal mutations in this region among PC. More sample verification is required. For PC patients with wild-type or mutant UTX, the prognosis and therapeutic effect of gemcitabine are unclear and will be our future research focus. What’s more, Another study revealed that the depletion of UTX increased the expression of TP63 in PC, a vital member of the p53 family of transcription factors that are instrumental in regulating cell cycle progression, apoptosis, and DNA repair processes (Andricovich et al. 2018). Further investigation is necessary to understand how G0S2, the target gene of our study, and transcription factors such as TP63, influence the progression of PC.

## Conclusion

This work revealed a novel mechanism by which the missense mutation on the JmjC domain of UTX impaired its antitumor effect in PC. Mechanistically, the mutation of UTX partially lost its histone H3K27 demethylase and elevated the H3K27me3 modification level of the G0S2 promoter, which decreased its expression and promoted the Toll-like/MAPK signaling pathway to weaken the antitumor effect of wild-type UTX in pancreatic carcinoma.

## Supplementary Information

Additional file 1. Figure S1. Knockdown G0S2 in PANC-1 cells with UTX-Wt overexpression reversed the tumor-suppressing capability of UTX-Wt. Construction of G0S2 knockdown using siRNA in PANC-1 cells. When PANC-1 cells grew to a certain degree of fusion, they were transfected with UTX-Wt overexpression plasmid, G0S2 knockdown plasmid, and their combined transfection for follow-up experiments. In addition, PANC-1 cells transfected with pCDH + siR-NC were used as negative controls. A WB assay was performed to validate the efficiency of silenced G0S2 in PANC-1 cells. After the knockdown of G0S2 in wild-type UTX-overexpressed PANC-1 cells, the abilities of invasiveness and migration, clone, and proliferationwere examined by Transwell, clone assays, and MTT, respectively. All data came from at least three repeated experiments. *P < 0.05, **P < 0.01.

## Data Availability

No datasets were generated or analysed during the current study.
