# Supplementary figures and images for: Mutation on JmjC domain of UTX impaired its antitumor effects in pancreatic cancer via inhibiting G0S2 expression and activating the Toll-like signaling pathway
Source: Mol Med. 2024 Dec 20;30:258. doi: 10.1186/s10020-024-01023-2 (PMC11660670; doi:10.1186/s10020-024-01023-2)

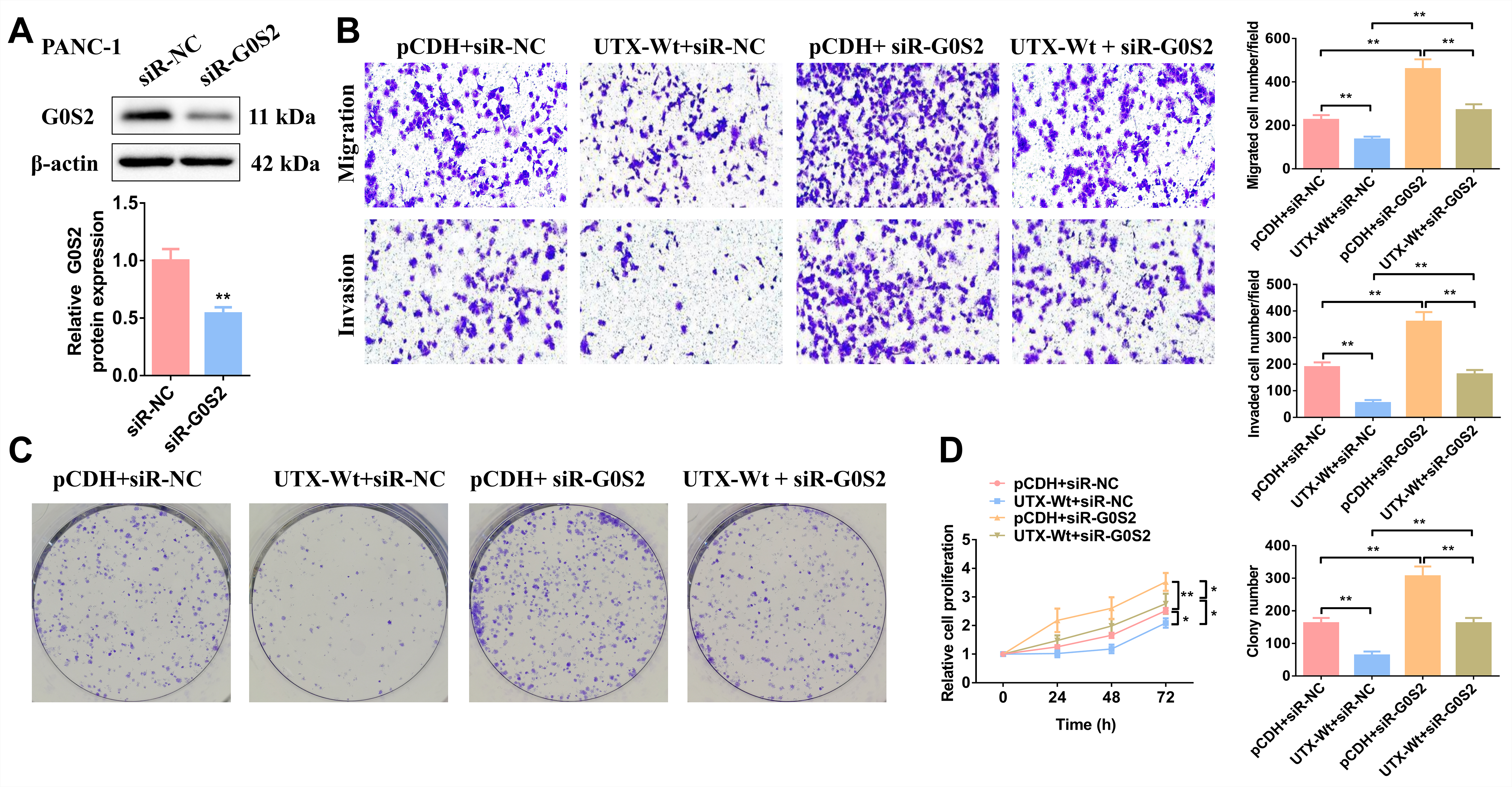

Supplement: Supplementary file 1 — Additional file 1. Figure S1. Knockdown G0S2 in PANC-1 cells with UTX-Wt overexpression reversed the tumor-suppressing capability of UTX-Wt. Construction of G0S2 knockdown using siRNA in PANC-1 cells. When PANC-1 cells grew to a certain degree of fusion, they were transfected with UTX-Wt overexpression plasmid, G0S2 knockdown plasmid, and their combined transfection for follow-up experiments. In addition, PANC-1 cells transfected with pCDH + siR-NC were used as negative controls. A WB assay was performed to validate the efficiency of silenced G0S2 in PANC-1 cells. After the knockdown of G0S2 in wild-type UTX-overexpressed PANC-1 cells, the abilities of invasiveness and migration, clone, and proliferationwere examined by Transwell, clone assays, and MTT, respectively. All data came from at least three repeated experiments. *P < 0.05, **P < 0.01. [file 10020_2024_1023_MOESM1_ESM.tif]
